# Bibliography

**Published:** 1899-01

**Authors:** 


### Bibliography.

Anatomy and Histology of the Mouth and Teeth. By I. Norman Broomell, D.D.S., Professor of Dental Anatomy, Dental Histology, and Prosthetic Technics in the Pennsylvania College of Dental Surgery, Philadelphia. With two hundred and eighty-four illustrations. P. Blakiston, Son & Co., Philadelphia, 1898.

When an author gives to the world original work he should receive a warm welcome. The dental profession has had book upon book thrust upon it with no pretence of presenting more than a compilation of the work of others, so that a feeling of dread comes over the reviewer when forced to give an honest opinion of the merit of any production.

It is, therefore, refreshing to take up this work of Dr. Broomell, to find that he has not been satisfied with the work of other men, but has gone to the only source of information, wherever the teeth are concerned, the tissues as they are developed. In this way he disarms criticism, for whoever calls in question his conclusions must adopt the same methods and secure proof from the same source.

So accustomed has the average observer become to forms of teeth shaped by the tool of the engraver that it will be difficult for some to accept these presented from photographs and in half-tone pictures, as really representing typical forms, but that they do represent teeth as they are and not as usually carefully drawn cannot be disputed. The only objection that can be made is in the selection of typical forms. This is always difficult, but it is thought that a better central incisor might have been secured than the one represented on page 133 with its abnormal crown and stumpy root.

The author has devoted the first part of this book, comprising eighty-eight pages, to what he terms "a gross description of the mouth and those tissues which enter into its construction." It is thought that the work would have been more satisfactory had this been omitted. The tendency of some writers to insert matter that should properly be kept within special lines cannot be commended. The subjects treated within the pages mentioned would appear better in works of anatomy, and it is from these most students would prefer to gain their knowledge.

The attention of the author is called to the illustration on page 78, in which the period of full development of the deciduous teeth is termed "youth." This is a broad term, too broad to be used to classify a period which strictly should be named childhood.

The real work of the author begins on page 89, Chapter IV., under the title of "A General Description of the Teeth." This is thorough and well arranged for study. In this general description the author states "that the superior first bicuspid may have one or two roots, most frequently the latter, while in the second bicuspid a single root is generally present." No allusion is here made to the very frequent presentation of three roots on the first. On page 171 there is a slight mention of this fact, but that it is not infrequent, should, it would seem, be mentioned. No doubt the author regarded this as an abnormality; if so, then a separate chapter might, with profit, have been devoted to these peculiar forms, which in many cases seem to present a tendency to reversion to original types.

In Chapter VIII. the reader is presented with the beginning of Dr. Broomell's most satisfactory work. The picture placed at the head of the page must attract the student and tells its own story, and at the same time it demonstrates the painstaking care of the author. The reviewer is not aware of any work to be compared with this, illustrating, as it does, by photograph the beginning of calcification in a central incisor. This is shown first in the cutting edge, and then by regular steps up through half crown, whole crown, to the final completion of the root. The text fully explains this in detail, covering all surfaces in the description. This method is followed throughout the entire series. First pictures of calcification, then the teeth in detail, with differences in various temperaments.

It is a question whether photography is equal to the presentation of the occlusal surfaces of molars. The depressions and fissures do not come out well, and seem to present deep cavities that destroy the harmonious lines natural to the surface. This is particularly noticeable on page 187, in the illustration of the occlusal surface of the superior second molar, and is in degree true of all the representations of this surface of the molar teeth. The old methods of careful drawing will give results more satisfactory with this surface.

The author does not carry out the calcification idea with the inferior teeth. This would, of course, have been an unnecessary labor, as the development here begins and ends at practically the same periods. The same care is observed, as to details, with these as with the superior set. One hundred and one pages are devoted to this portion of the work, involving an amount of careful labor rarely met with in books of this character.

From the consideration of these forms the reader is taken to the "Pulp-Cavities of the Teeth." These are treated in a similar manner. First the central incisor from the sixth to the tenth year, with the various changes from the formation of the pulp-chamber to the completion of the pulp-canal in the perfected root. This method is carried through all the teeth, very vividly reproducing the pulp-canals as they really are, all of them being made from dissections of natural teeth.

The deciduous teeth are then fully described from photographic pictures.

The chapter on "Development of the Teeth" begins a portion of the work to which the author has devoted a great amount of original research. His work upon the earlier stages of development is not as full as some would like to have it, but the author preferred "to treat the general subject from a macroscopic rather than a microscopic stand-point." The dissections made furnish a complete description of the visible development of tooth follicles. The reviewer is not familiar with any book that can pretend to show better work or give anything approaching the satisfactory character of these illustrations, and yet they do not give full credit to the original photographs, which the reviewer had the privilege of examining. While the illustrations naturally arrest the reader's attention, it would be an injustice to the author to omit an expression of satisfaction with the text. It follows the facts, as gained by dissection, with a clearness of description and faithfulness to results worthy of special commendation.

The balance of the book, from page 325 to 419, is devoted to histology, general and special, including the tissues of the teeth, in which Williams and Gysi are drawn upon for illustrations to considerable extent in connection with the original work of the author.

When a second edition is being prepared some things might be omitted with advantage, confining the work mainly to the author's own investigations, and, as before stated, a chapter on abnormalities, many-rooted teeth, supernumeraries, and twin teeth might with great profit to the student be added. In pulp-canals illustrations might be introduced showing variations from the normal opening at the apical foramen.

With Dr. Black's anatomy and this of Dr. Broomell the dental profession possesses two books that practically exhaust the subjects treated, and leave little to be desired as text-books in dental colleges or for the instruction of those more advanced.

The general make-up of this book is in every way worthy the house of P. Blakiston, Son & Co., being exceptionally good throughout.

LEHRBUCH DER CONSERVIRENDEN ZAHNHEILKUNDE. Von W. D. Miller, a. o., Professor an der Universität, Berlin. Mit 449 Abbildungen. Zweite umgearbeitete und erweiterte Auflage. Verlag von Georg Thieme. Leipzig, 1898.

This, the second edition in two years of this work of Professor Miller, indicates, more than words, the high appreciation in which it is held in Germany.

The author, in his introduction, has the idea, and it is doubtless true of continental Europe, that not one-fourth of practising dentists there care for the conservation of teeth, but prefer the preparation of artificial dentures. His aim has been to infuse into practitioners a stronger desire to save teeth by filling, and hence the large portion of this work is given up to this branch and collateral subjects.

The author wastes very little time in preliminaries, giving but fourteen pages to subjects by way of introduction to the second chapter on "The Filling of the Teeth." This very properly begins with the "Materials for Filling," and is thoroughly done, covering forty-four pages.

The author gives considerable space to the description of instruments, coffer-dam, etc.

From this point on is carried, step by step, through the process of preparing cavities, the handling of instruments, filling, etc.

The author's idea of the "Treatment of Hypersensitivity of Dentine" is fully described. His faith seems to be centred upon carbolic acid and cocaine, and gives the following formula:

℞ Acidi carbolici, 1.0;
Olei caryophylli, 1.0;
Cocaine hydrochlorate, 0.5.

Cataphoresis receives fair treatment, but the author does not seem to be favorably impressed with its value, preferring to give the experience of others.

The methods adopted in filling teeth are in nowise different from those understood and practised elsewhere, but all the processes are clearly stated and profusely illustrated.

"The Treatment of Diseased Pulps" follows in natural sequence. In regard to iodoform the author says, "Of all the remedies which have become practical in pulpitis I regard iodoform as the best."

For a paste to apply to a pulp the author makes use of the following:

℞ Acidi arseniosi,
Thymol,
Olei caryophylli, q. s.
ut ft. pasta.

In pyorrhœa alveolaris the author quotes all the great variety of names that have been attached to this disease, and very aptly suggests that it would be well to wait until we know the disease better before giving it a name.

In the subchapter on "The Bleaching of Teeth" the author gives, on page 422, the method of bleaching by chlorinated lime and acetic acid, but gives no credit to the originator of this process, which was by the writer of this review. No allusion would be made to this were it not the habit of nearly all dental writers to ignore the fact. It is presumed that Professor Miller, owing to long-continued illness, had very little to do with the final preparation of the book, hence the omission. For information the writer would state that the entire process of bleaching teeth, the preparation of the canal for the agent used, now universally adopted, instruments and method, originated as stated. The long, tedious experimentation that this entailed makes it worthy of some recognition. Bleaching teeth was regarded for twenty years or more by the majority of dentists as a fancy operation, and of no special value. When some more intelligent than the majority examined into the process and applied it, they received the credit, and the originator's part in it has been practically lost through one writer copying from another. There should be no difficulty in regard to this, as the whole matter was fully explained in the "American System of Dentistry." While it is true that the more modern methods have largely superseded this and have relegated the chlorine process to the background, it still retains a value for peculiar cases.

This book, as a whole, must be regarded as one of the best of its kind. The arrangement as a work on operative dentistry is very satisfactory. If there is any fault, it lies in too brief handling of the subjects, yet the book is quite large enough for convenient study, being 462 pages.

Unless this book should, some time in the future, be translated, it can have no value for English readers. Were this done, it would be a valuable addition to our text-books, but with several good books on this subject it is questionable whether it would find a demand sufficient to meet the outlay financially required for its preparation.

